# The genome sequence of the 16-spot Ladybird, *Tytthaspis sedecimpunctata* (Linnaeus, 1758) (Coleoptera: Coccinellidae)

**DOI:** 10.12688/wellcomeopenres.26880.1

**Published:** 2026-06-27

**Authors:** Liam M. Crowley, Olga Sivell, Ryan Mitchell, Duncan Sivell, Helen E. Roy, Peter M. J. Brown, University of Oxford and Wytham Woods Genome Acquisition Lab, Natural History Museum Genome Acquisition Lab, Darwin Tree of Life Barcoding Collective, Wellcome Sanger Institute Tree of Life Management, Samples and Laboratory team, Wellcome Sanger Institute Scientific Operations: Sequencing Operations, Wellcome Sanger Institute Tree of Life Core Informatics team, Tree of Life Core Informatics collective, Darwin Tree of Life Consortium

**Affiliations:** 1 University of Oxford, Oxford, England, UK; 2 Natural History Museum, London, England, UK; 3 Independent researcher, Sligo, County Sligo, Ireland; 4 UK Centre for Ecology & Hydrology, Wallingford, England, UK; 5 University of Exeter, Centre for Ecology and Conservation, Penryn, Cornwall, England, UK; 6 Anglia Ruskin University, Applied Ecology Research Group, School of Life Sciences, Cambridge, England, UK

**Keywords:** Tytthaspis sedecimpunctata, 16-spot Ladybird, genome sequence, chromosomal, Coleoptera

## Abstract

We present a genome assembly from an individual male *Tytthaspis sedecimpunctata* (16-spot Ladybird; Arthropoda; Insecta; Coleoptera; Coccinellidae). The genome sequence has a total length of 355.68 megabases. Most of the assembly (88.7%) is scaffolded into 10 chromosomal pseudomolecules, including the X sex chromosome. The mitochondrial genome has also been assembled, with a length of 18.38 kilobases. This assembly was generated as part of the Darwin Tree of Life project, which produces genomes for eukaryotic species found in Britain and Ireland.

## Species taxonomy

Eukaryota; Opisthokonta; Metazoa; Eumetazoa; Bilateria; Protostomia; Ecdysozoa; Panarthropoda; Arthropoda; Mandibulata; Pancrustacea; Altocrustacea; Allotriocarida; Hexapoda; Insecta; Dicondylia; Pterygota; Neoptera; Eumetabola; Endopterygota; Aparaglossata; Neuropteroidea; Coleoptera; Polyphaga; Cucujiformia; Coccinelloidea; Coccinellidae; Coccinellinae; Tytthaspidini; *Tytthaspis*; *Tytthaspis sedecimpunctata* (Linnaeus, 1758) (NCBI:txid347477).

## Background

The three fused spots creating a wiggly line at the base of the elytra are characteristic of the tiny (3 mm), beige-coloured 16-spot ladybird, *Tytthaspis sedecimpunctata.* The remaining five spots are distributed in an arc spanning the length of the elytra. A dark line marks the join of the elytra. This mildew-feeding ladybird is associated with grasslands but also common on scrub, heath and dunes ( Roy & Brown, 2018). The adults can form spectacular aggregations comprising hundreds of individuals within, as examples, cracks in fence posts or other sheltered crevices. The larvae are quite dull but do have long black hairs emerging from little bumps called tubercles.

The food preferences of ladybirds has been well-studied primarily because the predatory species have the potential to be used within biological control programmes. However, feeding relationships can reveal fascinating evolutionary perspectives and inform understanding of phylogenies ( Escalona *et al.*, 2017). Emergence of predatory life-styles are considered to be an important development in the evolutionary history of ladybirds with adaptations to herbivory, sporophagy and pollenphagy coming later. However, recent studies suggest that there have been repeated and phylogenetically independent transitions from coccid and aphid-feeding to other food preferences including fungal-feeding.

A recent study proposes two types of fungal-feeding ladybirds: specialized and obligate mycophagous species feeding on hymenium and conidia of powdery mildew fungi (Erysiphales) (e.g. *Halyzia* and *Psyllobora*) and more generalist species with a mixed diet including spores of various Ascomycete fungi but also plant tissue, pollen, acari and Thysanoptera (e.g *Tytthaspis*) ( Escalona *et al.*, 2017). The mandibles provide insights into the adaptations that assist these life-styles. As examples, specialist mildew feeders such as orange ladybirds, *Halyzia sedecimguttata*, have additional serration along the edges of the mandibles modified for scraping the micro-fungal structures from plant surfaces while 16-spot ladybirds have a stiff movable plate (prostheca) at the inner base of the mandible to scoop spores and pollen.

The dynamic evolutionary history of ladybirds with respect to food preferences has possibly led to misleading conclusions from phylogenies based on convergence of morphological traits because of the switching from feeding on coccids and aphids to fungal spores and plant tissues. The ephemeral nature of prey insects is considered to have presented opportunities for ladybirds to adapt to other foods, including micro-fungi and pollen, during times of limited prey. This adaptive capacity is thought to have played a major role in the diversification of ladybirds in some cases leading to specialisms to these alternative foods.

**Figure 1.**
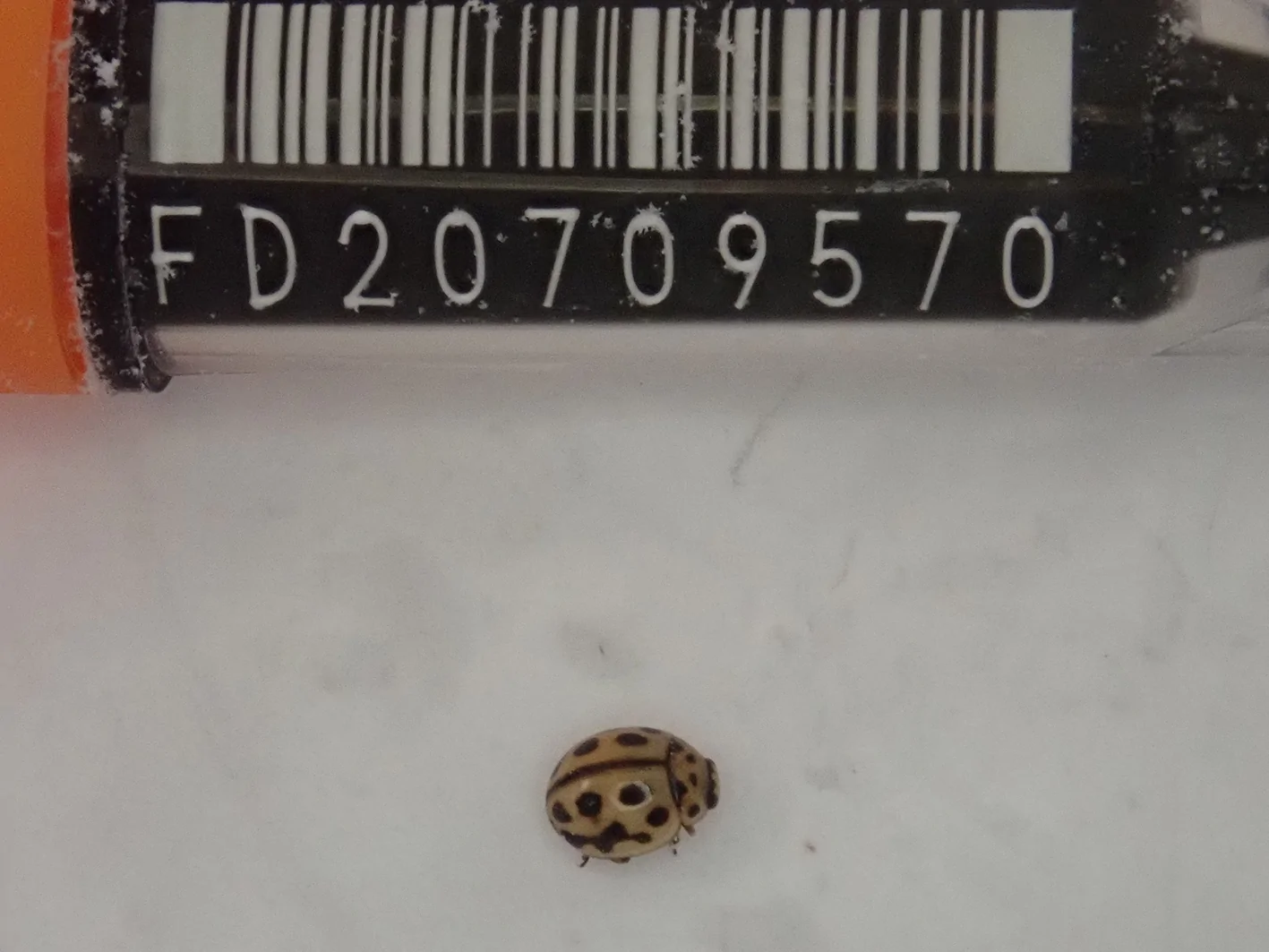
Photograph of the *Tytthaspis sedecimpunctata* (icTytSede3) specimen used for genome sequencing.

We present a chromosome-level genome sequence for *Tytthaspis sedecimpunctata*, the 16-spot Ladybird. The assembly was produced using the Tree of Life pipeline using a specimen collected from Wytham Farm, Berkshire, United Kingdom ( Figure 1). This assembly was generated as part of the Darwin Tree of Life Project, which aims to generate high-quality reference genomes for all named eukaryotic species in Britain and Ireland to support research, conservation, and the sustainable use of biodiversity ( Darwin Tree of Life Project Consortium, 2022).

## Methods

### Sample acquisition

The specimen used for genome sequencing was an adult male *Tytthaspis sedecimpunctata* (specimen ID Ox001185, ToLID icTytSede3; Figure 1), collected from Wytham Farm, Berkshire, United Kingdom (latitude 51.786, longitude −1.317) on 2021-04-13. The specimen was collected and identified by Liam Crowley. A second specimen was used for Hi-C sequencing (specimen ID NHMUK015059523, ToLID icTytSede6). It was collected from Morston Creek, Blakeney Estuary, England, UK (latitude 52.96, longitude −0.98) on 2022-07-07. The specimen was collected by Olga Sivell and identified by Ryan Mitchell. Another specimen was used for RNA sequencing (specimen ID NHMUK014036733, ToLID icTytSede4). It was collected from Dry Sandford Pit, England, UK (latitude 51.6932, longitude −1.3258) on 2021-06-19. The specimen was collected by Ryan Mitchell and Olga Sivell and identified by Duncan Sivell.

### Nucleic acid extraction

Detailed protocols for nucleic acid extraction developed at the Wellcome Sanger Institute (WSI) Tree of Life Core Laboratory are available on protocols.io ( Howard *et al.*, 2025). The icTytSede3 specimen was weighed and triaged to determine the appropriate extraction protocol. Tissue from the whole organism was homogenised by powermashing using a PowerMasher II tissue disruptor. High molecular weight (HMW) DNA was extracted using the Automated MagAttract v2 protocol. DNA was sheared into an average fragment size of 12–20 kb following the Megaruptor®3 for LI PacBio protocol. Sheared DNA was purified by manual SPRI (solid-phase reversible immobilisation). The concentration of the sheared and purified DNA was assessed using a Nanodrop spectrophotometer and Qubit Fluorometer using the Qubit dsDNA High Sensitivity Assay kit. Fragment size distribution was evaluated by running the sample on the FemtoPulse system.

RNA was extracted from tissue of the icTytSede4 specimen in the Tree of Life Laboratory at the WSI using the RNA Extraction: Automated MagMax™ *mir*Vana protocol. The RNA concentration was assessed using a Nanodrop spectrophotometer and a Qubit Fluorometer using the Qubit RNA Broad-Range Assay kit. Analysis of the integrity of the RNA was done using the Agilent RNA 6000 Pico Kit and Eukaryotic Total RNA assay.

### PacBio HiFi library preparation and sequencing

Library preparation and sequencing were performed at the WSI Scientific Operations core. Libraries were prepared using the SMRTbell Prep Kit 3.0 (Pacific Biosciences, California, USA), following the manufacturer’s instructions. The kit includes reagents for end repair/A-tailing, adapter ligation, post-ligation SMRTbell bead clean-up, and nuclease treatment. Size selection and clean-up were performed using diluted AMPure PB beads (Pacific Biosciences). DNA concentration was quantified using a Qubit Fluorometer v4.0 (ThermoFisher Scientific) and the Qubit 1X dsDNA HS assay kit. Final library fragment size was assessed with the Agilent Femto Pulse Automated Pulsed Field CE Instrument (Agilent Technologies) using the gDNA 55 kb BAC analysis kit.

The sample was sequenced using the Sequel IIe system (Pacific Biosciences, California, USA). The concentration of the library loaded onto the Sequel IIe was in the range 40–135 pM. SMRT Link software, a PacBio web-based workflow manager, was used to set up and monitor the run, and to perform primary and secondary analysis of the data upon completion.

### Hi-C

***Sample preparation and crosslinking***

The Hi-C sample was prepared from 20–50 mg of frozen tissue from the icTytSede6 sample using the Arima-HiC v2 kit (Arima Genomics). Following the manufacturer’s instructions, tissue was fixed and DNA crosslinked using TC buffer to a final formaldehyde concentration of 2%. The tissue was homogenised using the Diagnocine Power Masher-II. Crosslinked DNA was digested with a restriction enzyme master mix, biotinylated, and ligated. Clean-up was performed with SPRISelect beads before library preparation. DNA concentration was measured with the Qubit Fluorometer (Thermo Fisher Scientific) and Qubit HS Assay Kit. The biotinylation percentage was estimated using the Arima-HiC v2 QC beads.

***Hi-C library preparation and sequencing***

Biotinylated DNA constructs were fragmented using a Covaris E220 sonicator and size selected to 400–600 bp using SPRISelect beads. DNA was enriched with Arima-HiC v2 kit Enrichment beads. End repair, A-tailing, and adapter ligation were carried out with the NEBNext Ultra II DNA Library Prep Kit (New England Biolabs), following a modified protocol where library preparation occurs while DNA remains bound to the Enrichment beads. Library amplification was performed using KAPA HiFi HotStart mix and a custom Unique Dual Index (UDI) barcode set (Integrated DNA Technologies). Depending on sample concentration and biotinylation percentage determined at the crosslinking stage, libraries were amplified with 10–16 PCR cycles. Post-PCR clean-up was performed with SPRISelect beads. Libraries were quantified using the AccuClear Ultra High Sensitivity dsDNA Standards Assay Kit (Biotium) and a FLUOstar Omega plate reader (BMG Labtech).

Prior to sequencing, libraries were normalised to 10 ng/μL. Normalised libraries were quantified again to create equimolar and/or weighted 2.8 nM pools. Pool concentrations were checked using the Agilent 4200 TapeStation (Agilent) with High Sensitivity D500 reagents before sequencing. Sequencing was performed using paired-end 150 bp reads on the Illumina NovaSeq X.

### RNA library preparation and sequencing

Libraries were prepared using the NEBNext® Ultra II Directional RNA Library Prep Kit for Illumina (New England Biolabs), following the manufacturer’s instructions. Poly(A) mRNA was isolated from 100 ng of total RNA using oligo (dT) beads, converted to cDNA, and uniquely indexed; 14 PCR cycles were performed. Libraries were prepared with an intended fragment size of 100–300 bp. Libraries were quantified, normalised, and pooled equimolarly to a final concentration of 2.8 nM before dilution to 150 pM for loading. Sequencing was carried out on the Illumina NovaSeq X, generating paired-end reads.

### Genome assembly

Prior to assembly of the PacBio HiFi reads, a database of *k*-mer counts ( *k* = 31) was generated from the filtered reads using FastK. GenomeScope2 ( Ranallo-Benavidez *et al.*, 2020) was used to analyse the *k*-mer frequency distributions, providing estimates of genome size, heterozygosity, and repeat content.

The HiFi reads were assembled using Hifiasm ( Cheng *et al.*, 2021) with the --primary option. Haplotypic duplications were identified and removed using purge_dups ( Guan *et al.*, 2020). The Hi-C reads ( Rao *et al.*, 2014) were mapped to the primary contigs using bwa-mem2 ( Vasimuddin *et al.*, 2019), and the contigs were scaffolded in YaHS ( Zhou *et al.*, 2023) with the --break option for handling potential misassemblies. The scaffolded assemblies were evaluated using Gfastats ( Formenti *et al.*, 2022), BUSCO ( Manni *et al.*, 2021) and MerquryFK ( Rhie *et al.*, 2020).

The mitochondrial genome was assembled using MitoHiFi ( Uliano-Silva *et al.*, 2023). The MitoHiFi reference was the mitochondrial genome of *Coccinella transversoguttata* (NC_067078.1).

### Assembly curation

The assembly was decontaminated using the Assembly Screen for Cobionts and Contaminants ( ASCC) pipeline. TreeVal was used to generate the flat files and maps for use in curation. Manual curation was conducted primarily in PretextView and HiGlass ( Kerpedjiev *et al.*, 2018). Scaffolds were visually inspected and corrected as described by Howe *et al*. (2021). Manual corrections included 53 breaks and 47 joins. This reduced the scaffold count by 6.3% and reduced the scaffold N50 by 4.8%. The curation process is described at https://gitlab.com/wtsi-grit/rapid-curation . PretextSnapshot was used to generate a Hi-C contact map of the final assembly.

### Assembly quality assessment

The MerquryFK tool ( Rhie *et al.*, 2020) was run in a Singularity container ( Kurtzer *et al.*, 2017) to evaluate *k*-mer completeness and assembly quality for the primary and alternate haplotypes using the *k*-mer database ( *k* = 31) computed prior to genome assembly. The analysis outputs included assembly QV scores and completeness statistics.

The genome was analysed using the BlobToolKit pipeline, a Nextflow implementation of the earlier Snakemake version ( Challis *et al.*, 2020). The pipeline aligns PacBio reads using minimap2 ( Li, 2018) and SAMtools ( Danecek *et al.*, 2021) to generate coverage tracks. It runs BUSCO ( Manni *et al.*, 2021) using lineages identified from the NCBI Taxonomy ( Schoch *et al.*, 2020). For the three domain-level lineages, BUSCO genes are aligned to the UniProt Reference Proteomes database ( Bateman *et al.*, 2023) using DIAMOND blastp ( Buchfink *et al.*, 2021). The genome is divided into chunks based on the density of BUSCO genes from the closest taxonomic lineage, and each chunk is aligned to the UniProt Reference Proteomes database with DIAMOND blastx. Sequences without hits are chunked using seqtk and aligned to the NT database with blastn ( Altschul *et al.*, 1990). The BlobToolKit suite consolidates all outputs into a blobdir for visualisation. The BlobToolKit pipeline was developed using nf-core tooling ( Ewels *et al.*, 2020) and MultiQC ( Ewels *et al.*, 2016), with containerisation through Docker ( Merkel, 2014) and Singularity ( Kurtzer *et al.*, 2017).

## Genome sequence report

### Sequence data

**Figure 2.**
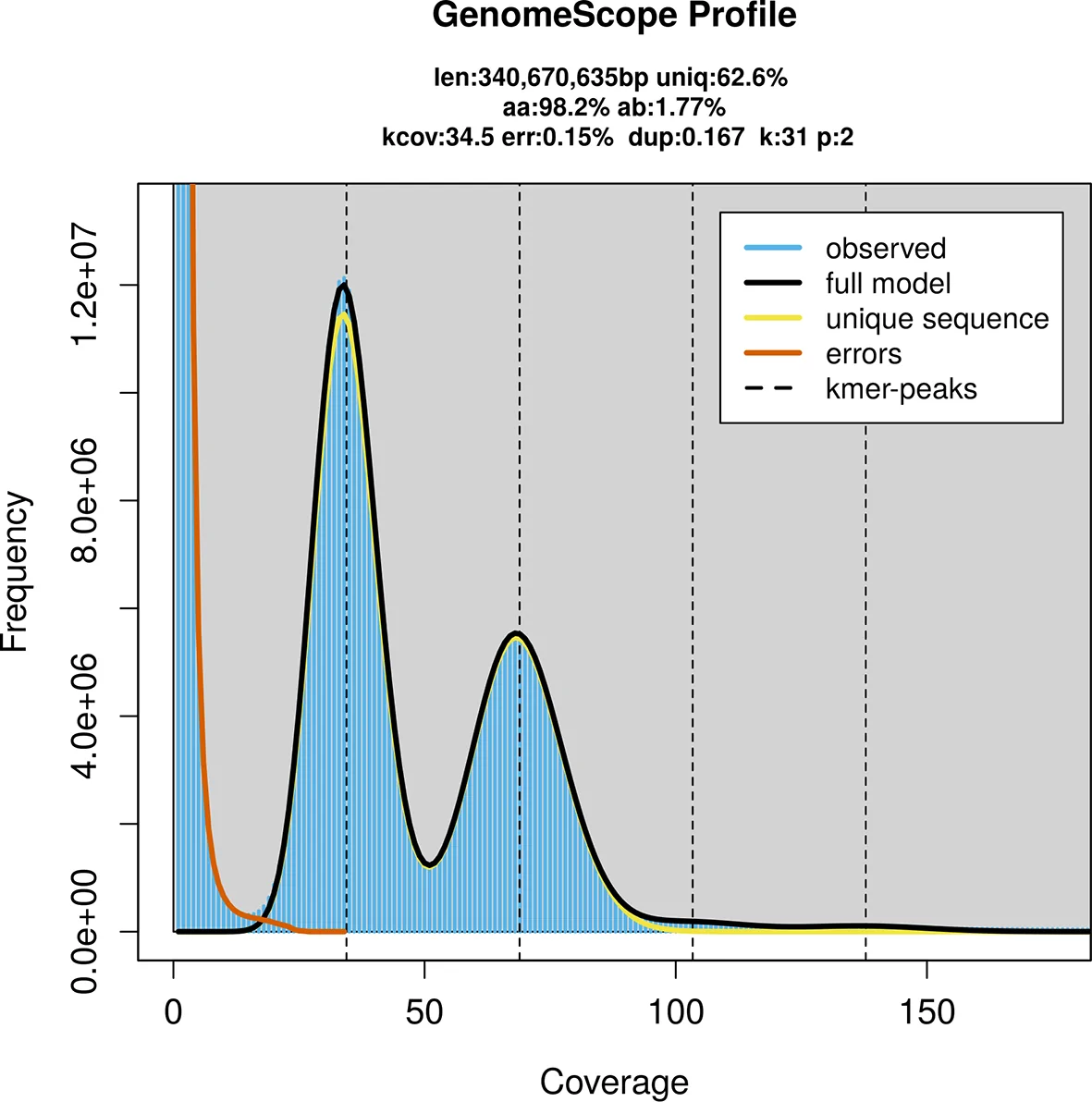
Frequency distribution of *k*-mers generated using GenomeScope2. The plot shows observed and modelled *k*-mer spectra, providing estimates of genome size, heterozygosity, and repeat content based on unassembled sequencing reads.

PacBio sequencing of the *Tytthaspis sedecimpunctata* specimen generated 24.66 Gb (gigabases) from 2.19 million reads, which were used to assemble the genome. GenomeScope2.0 analysis estimated the haploid genome size at 340.93 Mb, with a heterozygosity of 1.77% and repeat content of 37.37% ( Figure 2). These estimates guided expectations for the assembly. Based on the estimated genome size, the sequencing data provided approximately 69× coverage. Hi-C sequencing produced 287.65 Gb from 952.49 million reads, which were used to scaffold the assembly. RNA sequencing data were also generated and are available in public sequence repositories. Table 1 summarises the specimen and sequencing details.

**Table 1.** Specimen and sequencing data for *Tytthaspis sedecimpunctata* (BioProject PRJEB73900).

Platform	PacBio HiFi	Hi-C	RNA-seq
**ToLID**	icTytSede3	icTytSede6	icTytSede4
**Specimen ID**	Ox001185	NHMUK015059523	NHMUK014036733
**BioSample (source individual)**	SAMEA10107102	SAMEA112964274	SAMEA11025073
**BioSample (tissue)**	SAMEA10200828	SAMEA112975443	SAMEA11025328
**Tissue**	whole organism	whole organism	other somatic animal tissue
**Instrument**	Sequel IIe	Illumina NovaSeq X	Illumina NovaSeq X
**Run accessions**	ERR12760795	ERR12765172; ERR12765173	ERR13093654
**Read count total**	2.19 million reads	952.49 million read pairs	84.29 million read pairs
**Base count total**	24.66 Gb	287.65 Gb	25.45 Gb

### Assembly statistics

The primary haplotype was assembled, and contigs corresponding to an alternate haplotype were also deposited in INSDC databases. The final assembly has a total length of 355.68 Mb in 73 scaffolds, with 68 gaps, and a scaffold N50 of 32.13 Mb ( Table 2).

**Table 2.** Genome assembly data for *Tytthaspis sedecimpunctata.*

Genome assembly	Primary assembly
**Assembly name**	icTytSede3.1
**Assembly accession**	GCA_977970285.1
**Alternate haplotype accession**	GCA_977970265.1
**Assembly level**	chromosome
**Span (Mb)**	355.68
**Number of chromosomes**	10
**Number of contigs**	141
**Contig N50**	8.95 Mb
**Number of scaffolds**	73
**Scaffold N50**	32.13 Mb
**Sex chromosomes**	X
**Organelles**	Mitochondrial genome: 18.38 kb

**Figure 3.**
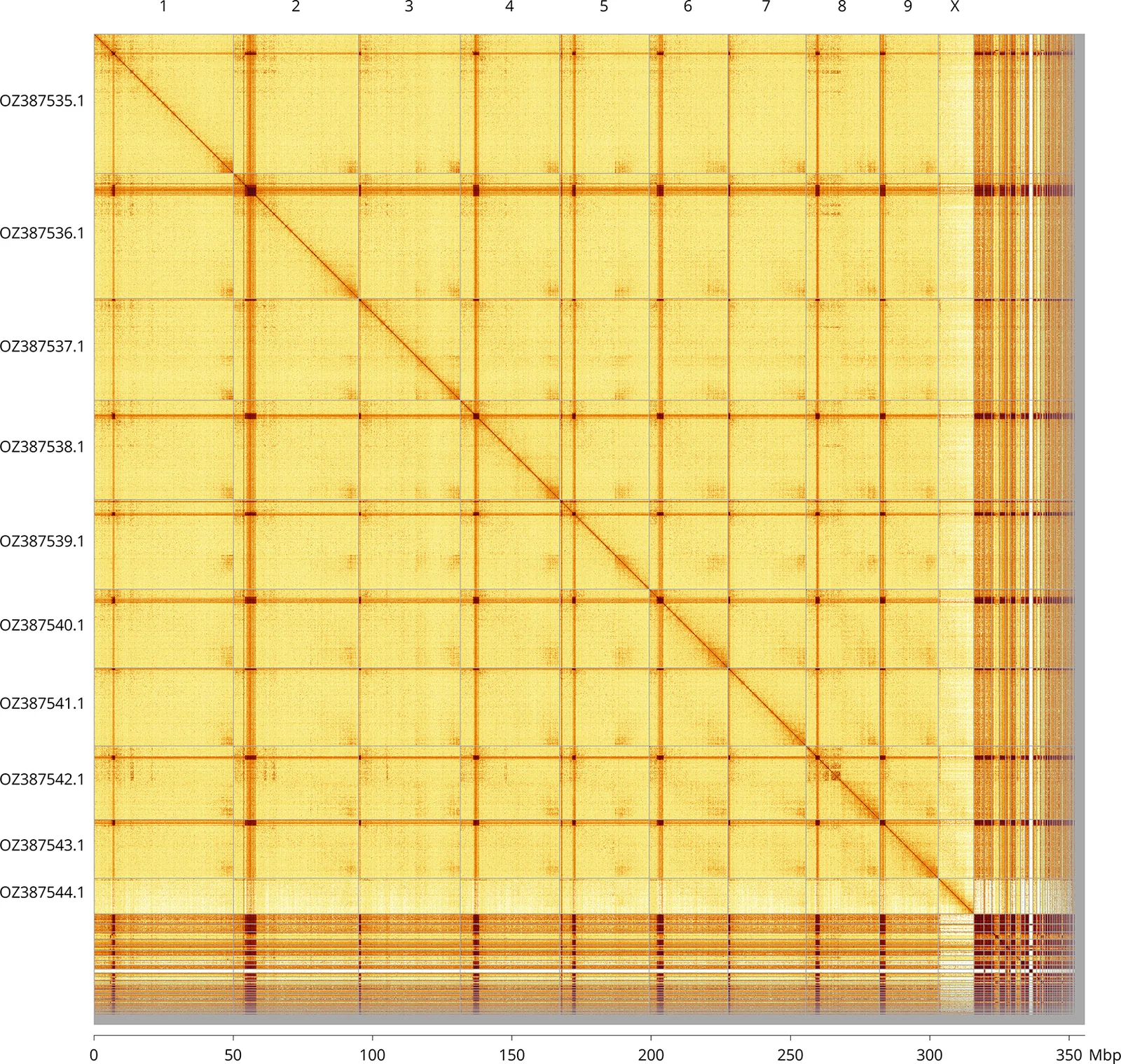
Hi-C contact map of the *Tytthaspis sedecimpunctata* genome assembly. Assembled chromosomes are shown in order of size and labelled along the axes, with a megabase scale shown below. The plot was generated using PretextSnapshot.

Most of the assembly sequence (88.7%) was assigned to 10 chromosomal-level scaffolds, representing 9 autosomes and the X sex chromosome. These chromosome-level scaffolds, confirmed by Hi-C data, are named according to size ( Figure 3; Table 3). X chromosome identified based on BUSCO gene painting using ancestral BUSCO genes. The Y chromosome could not be confidently identified in the assembled genome. A large number of centromeric repeats is in the unplaced sequences. The exact order and orientation of the contigs are unknown for the following chromosome intervals: chromosome 1: 5 813–7 566 Kbp, chromosome 2: 1–781 Kbp, chromosome 3: 5 813–7 566 Kbp, chromosome 4: 4 369–6 665 Kbp, chromosome 5: 4 461–5 688 Kbp, chromosome 6: 2 605–5 031 Kbp, chromosome 7: 1–754 Kbp, chromosome 8: 3 300–4 885 Kbp, and chromosome 9: 1–2 013 Kbp.

**Table 3.** Chromosomal pseudomolecules in the primary genome assembly of *Tytthaspis sedecimpunctata* icTytSede3.

INSDC accession	Molecule	Length (Mb)	GC%
OZ387535.1	1	50.09	35
OZ387536.1	2	44.83	35
OZ387537.1	3	36.49	35
OZ387538.1	4	35.63	35
OZ387539.1	5	32.13	35.50
OZ387540.1	6	28.16	35.50
OZ387541.1	7	28.04	35.50
OZ387542.1	8	26.38	36.50
OZ387543.1	9	20.99	36.50
OZ387544.1	X	12.75	36

The mitochondrial genome was also assembled (length 18.38 kb, OZ387545.1). This sequence is included as a contig in the multifasta file of the genome submission and as a standalone record.

### Assembly quality metrics

**Figure 4.**
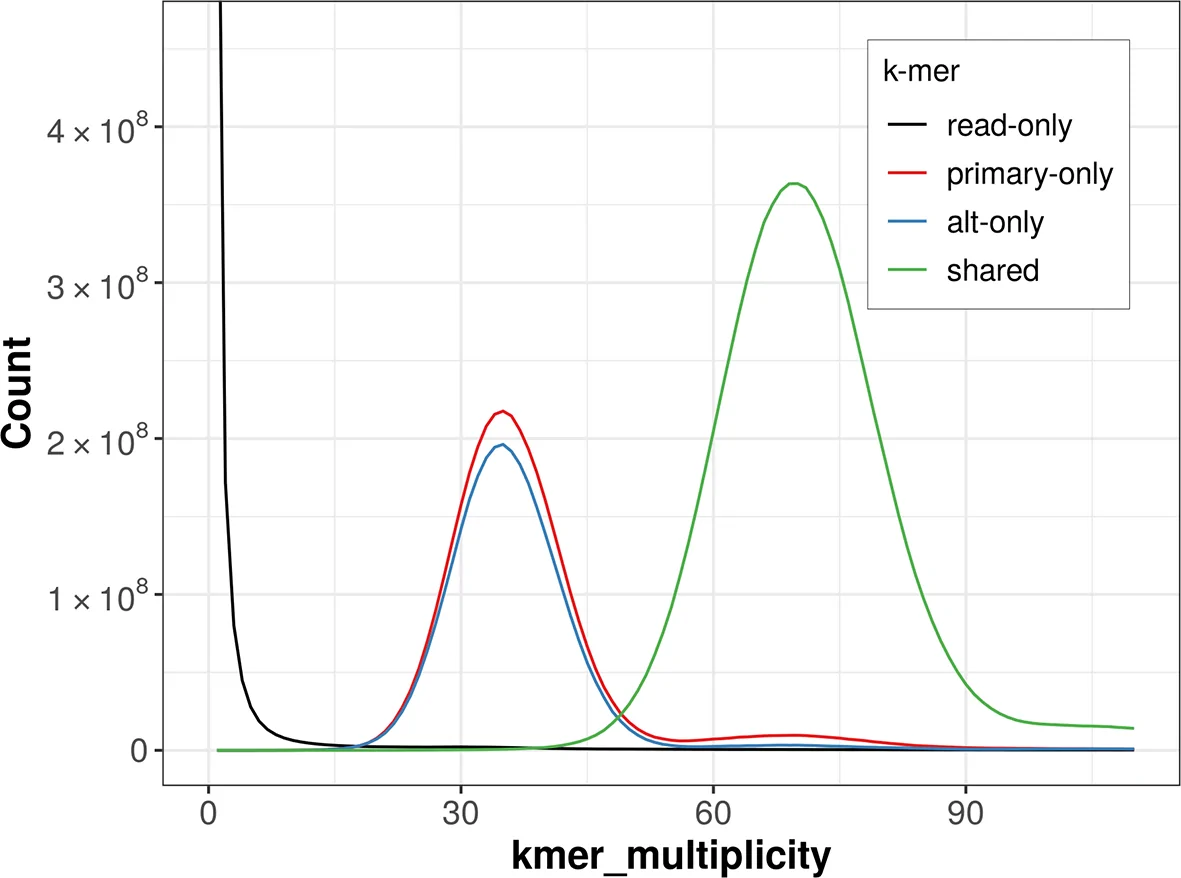
Evaluation of *k*-mer completeness using MerquryFK. This plot illustrates the recovery of *k*-mers from the original read data in the final assemblies. The horizontal axis represents *k*-mer multiplicity, and the vertical axis shows the number of *k*-mers. The black curve represents *k*-mers that appear in the reads but are not assembled. The green curve corresponds to *k*-mers shared by both haplotypes, and the red and blue curves show *k*-mers found only in one of the haplotypes.

The combined primary and alternate assemblies achieve an estimated QV of 63.3. The *k*-mer completeness is 72.10% for the primary assembly, 68.06% for the alternate haplotype, and 99.15% for the combined assemblies ( Figure 4).

**Figure 5.**
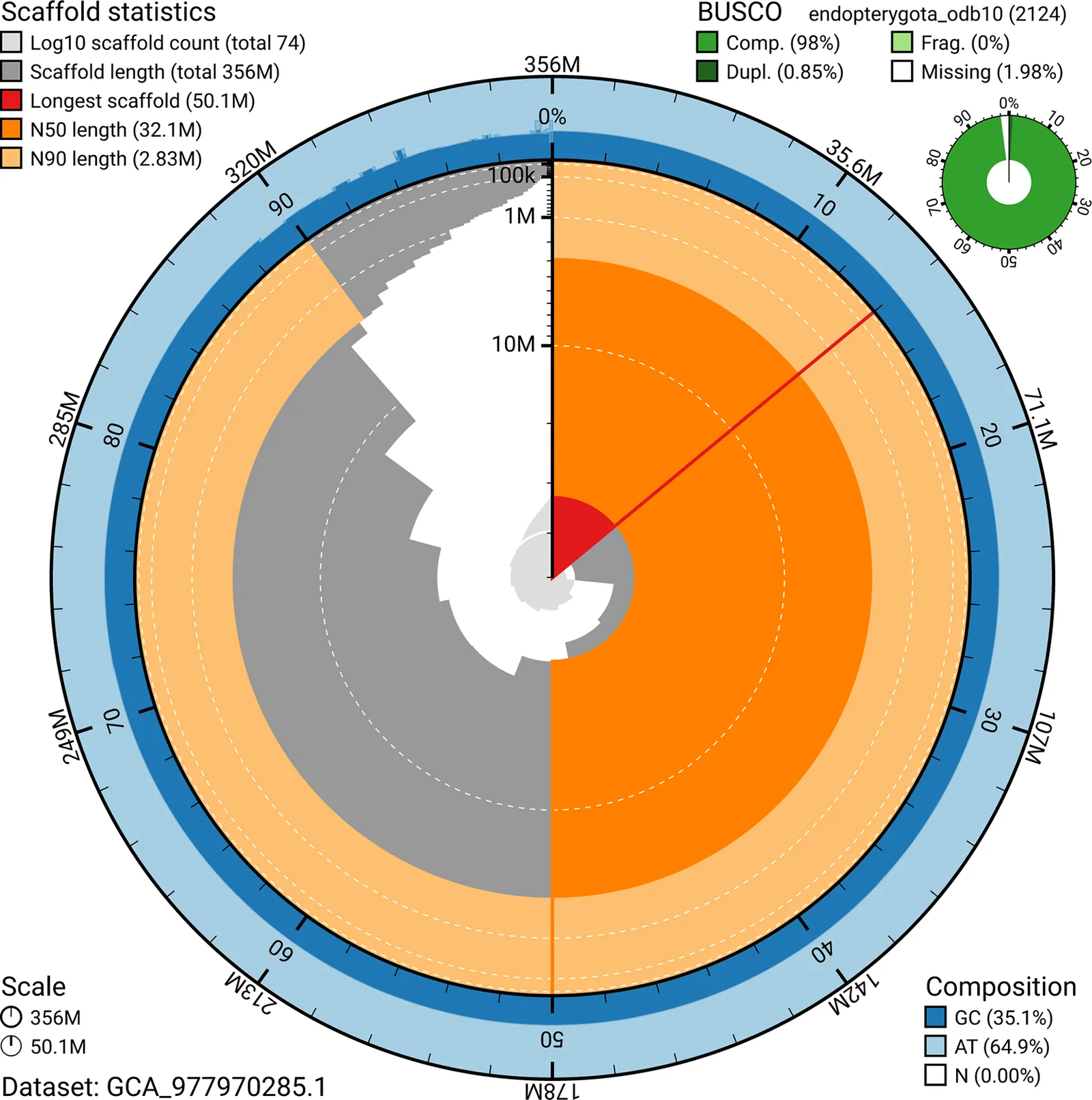
Assembly metrics for icTytSede3.1. The BlobToolKit snail plot provides an overview of assembly metrics and BUSCO gene completeness. The circumference represents the length of the whole genome sequence, and the main plot is divided into 1 000 bins around the circumference. The outermost blue tracks display the distribution of GC, AT, and N percentages across the bins. Scaffolds are arranged clockwise from longest to shortest and are depicted in dark grey. The longest scaffold is indicated by the red arc, and the deeper orange and pale orange arcs represent the N50 and N90 lengths. A light grey spiral at the centre shows the cumulative scaffold count on a logarithmic scale. A summary of complete, fragmented, duplicated, and missing BUSCO genes in the endopterygota_odb10 set is presented at the top right. An interactive version of this figure can be accessed on the BlobToolKit viewer.

**Figure 6.**
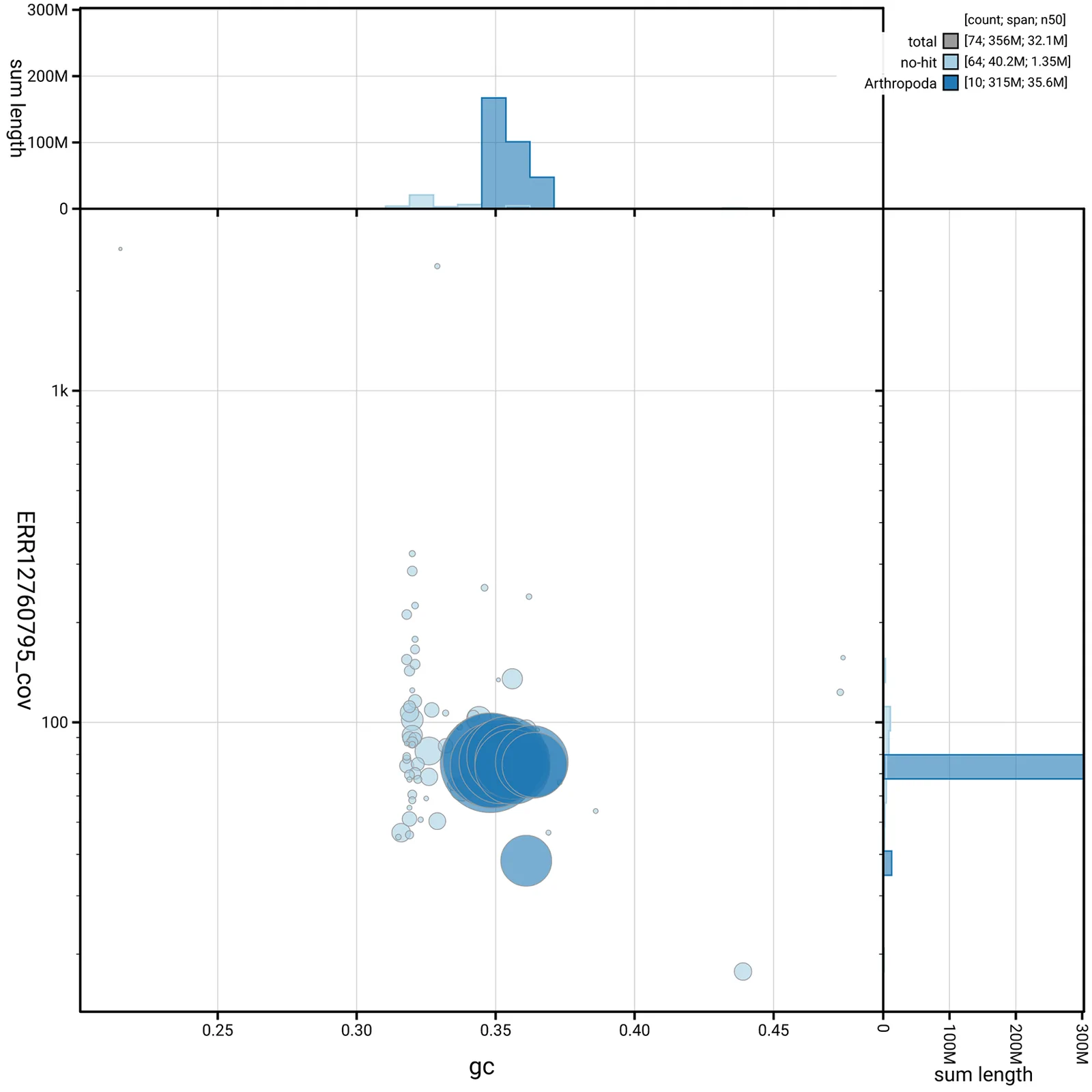
BlobToolKit blob plot for icTytSede3.1. The plot shows base coverage (vertical axis) and GC content (horizontal axis). The circles represent scaffolds, with the size proportional to scaffold length and the colour representing phylum membership. The histograms along the axes display the total length of sequences distributed across different levels of coverage and GC content. An interactive version of this figure is available on the BlobToolKit viewer.

BUSCO v.6.0.0 analysis using the endopterygota_odb10 reference set ( *n* = 2 124) identified 98.0% of the expected gene set (single = 97.2%, duplicated = 0.8%). The snail plot in Figure 5 summarises the scaffold length distribution and other assembly statistics for the primary assembly. The blob plot in Figure 6 shows the distribution of scaffolds by GC proportion and coverage.

Table 4 lists the assembly metric benchmarks adapted from Rhie *et al.* (2021) and the Earth BioGenome Project Report on Assembly Standards. The EBP metric, calculated for the primary assembly, is **6.7.Q64**.

**Table 4.** Earth Biogenome Project summary metrics for the *Tytthaspis sedecimpunctata* assembly.

Measure	Value	Benchmark
EBP summary (primary)	6.7.Q64	6.C.Q40
Contig N50 length	8.95 Mb	≥ 1 Mb
Scaffold N50 length	32.13 Mb	= chromosome N50
Consensus quality (QV)	Primary: 64.1; alternate: 62.9; combined: 63.3	≥ 40
*k*-mer completeness	Primary: 72.10%; alternate: 68.06%; combined: 99.15%	≥ 95%
BUSCO	C:98.0% [S:97.2%, D:0.8%], F:0.0%, M:2.0%, n:2 124	S > 90%; D < 5%
Percentage of assembly assigned to chromosomes	88.70%	≥ 90%

**Notes:** The EBP summary uses log10(Contig N50); chromosome-level (C) or log10(Scaffold N50); Q (Merqury QV). BUSCO: C = complete; S = single-copy; D = duplicated; F = fragmented; M = missing; n = orthologues.

**Table 5.** Software versions and sources used for *Tytthaspis sedecimpunctata.*

Software	Version	Source
BLAST	2.14.0	ftp://ftp.ncbi.nlm.nih.gov/blast/executables/blast+/
BlobToolKit	4.4.6	https://github.com/blobtoolkit/blobtoolkit
BUSCO	6.0.0	https://gitlab.com/ezlab/busco
bwa-mem2	2.2.1	https://github.com/bwa-mem2/bwa-mem2
DIAMOND	2.1.8	https://github.com/bbuchfink/diamond
fasta_windows	0.2.4	https://github.com/tolkit/fasta_windows
FastK	1.1	https://github.com/thegenemyers/FASTK
GenomeScope2.0	2.0.1	https://github.com/tbenavi1/genomescope2.0
Gfastats	1.3.6	https://github.com/vgl-hub/gfastats
Hifiasm	0.19.5-r587	https://github.com/chhylp123/hifiasm
HiGlass	1.13.4	https://github.com/higlass/higlass
MerquryFK	1.1.0-c1	https://github.com/thegenemyers/MERQURY.FK
Minimap2	2.24-r1122; 2.28-r1209	https://github.com/lh3/minimap2
MitoHiFi	2	https://github.com/marcelauliano/MitoHiFi
MultiQC	1.14; 1.17 and 1.18	https://github.com/MultiQC/MultiQC
Nextflow	23.10.0; 24.10.4	https://github.com/nextflow-io/nextflow
PretextSnapshot	0.0.4	https://github.com/sanger-tol/PretextSnapshot
PretextView	1.0.3	https://github.com/sanger-tol/PretextView
purge_dups	1.2.3	https://github.com/dfguan/purge_dups
samtools	1.17; 1.18; 1.2; 1.21; 1.6	https://github.com/samtools/samtools
sanger-tol/ascc	0.1.0	https://github.com/sanger-tol/ascc
sanger-tol/blobtoolkit	v0.9.0	https://github.com/sanger-tol/blobtoolkit
sanger-tol/curationpretext	1.4.2	https://github.com/sanger-tol/curationpretext
Seqtk	1.4-r122	https://github.com/lh3/seqtk
Singularity	3.9.0	https://github.com/sylabs/singularity
TreeVal	1.0.2	https://github.com/sanger-tol/treeval
YaHS	1.1a.2	https://github.com/c-zhou/yahs

## Author information

Contributors are listed at the following links:

• Members of the University of Oxford and Wytham Woods Genome Acquisition Lab
• Members of the Natural History Museum Genome Acquisition Lab
• Members of the Wellcome Sanger Institute Tree of Life Management, Samples and Laboratory team
• Members of Wellcome Sanger Institute Scientific Operations – Sequencing Operations
• Members of the Wellcome Sanger Institute Tree of Life Core Informatics team
• Members of the Tree of Life Core Informatics collective
• Members of the Darwin Tree of Life Consortium

## Wellcome Sanger Institute – Legal and Governance

The materials that have contributed to this genome note have been supplied by a Darwin Tree of Life Partner. The submission of materials by a Darwin Tree of Life Partner is subject to the **‘Darwin Tree of Life Project Sampling Code of Practice’**, which can be found in full on the Darwin Tree of Life website. By agreeing with and signing up to the Sampling Code of Practice, the Darwin Tree of Life Partner agrees they will meet the legal and ethical requirements and standards set out within this document in respect of all samples acquired for, and supplied to, the Darwin Tree of Life Project. Further, the Wellcome Sanger Institute employs a process whereby due diligence is carried out proportionate to the nature of the materials themselves, and the circumstances under which they have been/are to be collected and provided for use. The purpose of this is to address and mitigate any potential legal and/or ethical implications of receipt and use of the materials as part of the research project, and to ensure that in doing so we align with best practice wherever possible. The overarching areas of consideration are:

• Ethical review of provenance and sourcing of the material
• Legality of collection, transfer and use (national and international)

Each transfer of samples is further undertaken according to a Research Collaboration Agreement or Material Transfer Agreement entered into by the Darwin Tree of Life Partner, Genome Research Limited (operating as the Wellcome Sanger Institute), and in some circumstances, other Darwin Tree of Life collaborators.

## Data Availability

European Nucleotide Archive: Tytthaspis sedecimpunctata. Accession number PRJEB73900; https://identifiers.org/ena.embl/PRJEB73900. The genome sequence is released openly for reuse. The *Tytthaspis sedecimpunctata* genome sequencing initiative is part of the Darwin Tree of Life Project (PRJEB40665) and the Sanger Institute Tree of Life Programme (PRJEB43745). All raw sequence data and the assembly have been deposited in INSDC databases. The genome will be annotated using available RNA-Seq data and presented through the Ensembl pipeline at the European Bioinformatics Institute. Raw data and assembly accession identifiers are reported in Tables 1 and 2. Production code used in genome assembly at the WSI Tree of Life is available at https://github.com/sanger-tol . Table 5 lists software versions used in this study.
