# Supplementary material for: The genome sequence of the 16-spot Ladybird, Tytthaspis sedecimpunctata (Linnaeus, 1758) (Coleoptera: Coccinellidae)
Source: Wellcome Open Res. 2026 Jun 27;11:402. [Version 1] doi: 10.12688/wellcomeopenres.26880.1 (PMC13562938; doi:10.12688/wellcomeopenres.26880.1)
Supplement: Peer review history

# Reviewer report: Reviewer response for version 1

**DOI:** 10.21956/wellcomeopenres.29599.r164603

**Published:** 2026-09-03

**Authors:** Michael Hiller

**Affiliations:** 1 Senckenberg Research Institute, Frankfurt, Germany

This data note presents a high-quality assembly of the ladybird. The genome has a high contig N50, provides all autosomes and the X chromosome, has a high base accuracy and meets/exceeds all key assembly metrics. 

 The Y chromosome could not be identified and assembled, likely because it is too repeat-rich. 

 RNA-seq data was also generated and an annotation is forthcoming. 

 My only question is whether a karyotype of this species exists and if so, whether it matches the number of assembled chromosomes. Otherwise, I have no comments and recommend indexing the data note.

Are sufficient details of methods and materials provided to allow replication by others?

Yes

Is the rationale for creating the dataset(s) clearly described?

Yes

Are the datasets clearly presented in a useable and accessible format?

Yes

Are the protocols appropriate and is the work technically sound?

Yes

Reviewer Expertise:

genome assembly; comparative genomics

I confirm that I have read this submission and believe that I have an appropriate level of expertise to confirm that it is of an acceptable scientific standard.

# Reviewer report: Reviewer response for version 1

**DOI:** 10.21956/wellcomeopenres.29599.r164606

**Published:** 2026-09-01

**Authors:** Maria Antonia Madrid Restrepo

**Affiliations:** 1 KU Leuven, Leuven, Belgium

This data note presents a chromosome-level genome assembly of the male 16-spot ladybird, *T. sedecimpunctata*. PacBio HiFi and Hi-C data were used for assembly and scaffolding, with RNA-seq also deposited for future annotation. The 355.68 Mb assembly contains 73 scaffolds; 88.7% is assigned to nine autosomes and a proposed X chromosome. Reported quality is high and the raw reads, primary assembly, alternate haplotype, mitochondrial genome, and software versions are publicly available.

 The dataset contributes a high-quality reference genome to the Darwin ToL initiative and supports comparative, evolutionary, and conservation research.

 The sequencing, assembly, curation, contamination screening, and quality-assessment protocols are appropriate. The combination of HiFi, Hi-C, BUSCO, MerquryFK, and BlobToolKit provides adequate technical validation. The datasets are clearly identified through BioProject, BioSample, run, assembly, chromosome, alternate-haplotype, and mitochondrial accessions. 

 The assignment of the X chromosome is not well supported by the statement that it was identified through “BUSCO gene painting.” The authors must describe the analysis and evidence used for this assignment. Because the sequenced individual was male, they must also explain the failure to identify a Y chromosome and state the known or expected sex-determination system of this species, or of Coccinellidae if species-specific information is unavailable. They should distinguish biological absence from failure to assemble a repeat-rich or otherwise difficult Y chromosome.

Are sufficient details of methods and materials provided to allow replication by others?

Yes

Is the rationale for creating the dataset(s) clearly described?

Yes

Are the datasets clearly presented in a useable and accessible format?

Yes

Are the protocols appropriate and is the work technically sound?

Yes

Reviewer Expertise:

Evolutionary genomics

I confirm that I have read this submission and believe that I have an appropriate level of expertise to confirm that it is of an acceptable scientific standard.

# Reviewer report: Reviewer response for version 1

**DOI:** 10.21956/wellcomeopenres.29599.r160093

**Published:** 2026-07-24

**Authors:** Xuanzeng Liu

**Affiliations:** 1 College of Life Sciences, Shaanxi Normal University, Xi’an, China

Crowley et al. present a chromosome-level genome assembly for *Tytthaspis sedecimpunctata*. The assembly was generated using a standard and well-established workflow, and the overall assembly quality is good. The genomic data have been made publicly available, which will provide a valuable resource for future studies on ladybird biology, comparative genomics, and genome evolution. Overall, this is a high-quality genome data report, and I recommend publication in its current form after minor revision.

 I only have one minor suggestion for the authors.

 The authors state that the X chromosome was identified based on BUSCO gene painting using ancestral BUSCO genes, whereas the Y chromosome could not be confidently identified in the assembled genome. However, the manuscript does not discuss the possible reasons for the absence of an identifiable Y chromosome. It would be helpful if the authors could briefly explain whether this result is due to technical limitations or whether it could reflect the biology of the species.

 In addition, I suggest that the Introduction briefly describe the known sex determination system of *T. sedecimpunctata* or, more broadly, of Coccinellidae if species-specific information is unavailable. As a reader who is not a specialist in this group, it is unclear whether this species possesses an XX/XY, XX/XO, or another sex chromosome system. Providing this background would help readers better interpret the chromosome assignment and the apparent absence of the Y chromosome.

Are sufficient details of methods and materials provided to allow replication by others?

Yes

Is the rationale for creating the dataset(s) clearly described?

Yes

Are the datasets clearly presented in a useable and accessible format?

Yes

Are the protocols appropriate and is the work technically sound?

Yes

Reviewer Expertise:

genome evolution

I confirm that I have read this submission and believe that I have an appropriate level of expertise to confirm that it is of an acceptable scientific standard.
